# Endoscopic trans-orbital approach for the tumor-related epilepsy at the temporal tip

**DOI:** 10.3171/2024.4.FOCVID2414

**Published:** 2024-07-01

**Authors:** Yuta Tanoue, Takehiro Uda, Toshiyuki Kawashima, Vich Yindeedej, Takeo Goto

**Affiliations:** Department of Neurosurgery, Osaka Metropolitan University Graduate School of Medicine, Osaka, Japan

**Keywords:** endoscopic surgery, transorbital approach, minimally invasive surgery, epilepsy surgery, tumor-related epilepsy

## Abstract

Minimally invasive surgery is gaining increasing interest in epilepsy surgery. In this video, the authors present the endoscopic transorbital approach for an epileptogenic lesion located at the temporal tip. The patient was a man in his 40s who has had intractable focal impaired awareness seizures and focal to bilateral tonic-clonic seizures since he was 31 years of age. According to the preoperative examination, including stereotactic electroencephalography, a cavernous angioma located at the tip of the right temporal lobe was diagnosed as an epileptogenic lesion. Lesionectomy for this lesion was performed using the endoscopic transorbital approach as minimally invasive surgery and a favorable outcome was achieved.

The video can be found here: https://stream.cadmore.media/r10.3171/2024.4.FOCVID2414

## Transcript

Endoscopic transorbital approach for tumor-related epilepsy at the temporal tip.

### 0:26 Introduction.

Minimally invasive surgery is gaining increasing interest in epilepsy surgery, particularly following the introduction of stereotactic electroencephalography (SEEG).^1,2^ Additionally, endoscopic surgery has been expanding as minimally invasive procedure in neurosurgery.^3^ We previously reported endoscopic transcortical selective amygdalohippocampectomy and endoscopic corpus callosotomy as promising minimally invasive epilepsy surgeries.^4,5^ In this report, we present the endoscopic transorbital approach for an epileptogenic lesion located at the temporal tip.

### 1:08 Case Presentation.

The patient was a man in his 40s with intractable focal impaired awareness seizures and focal to bilateral tonic-clonic seizures since the age of 31 years. Scalp EEG showed bilateral temporal spikes at F7, T3, F8, and T4. MRI showed a cavernous angioma at the tip of the right temporal lobe with no obvious hippocampal signal change or atrophy. The patient had no neurological or cognitive impairments other than epilepsy.

### 1:42 Stereotactic Electroencephalography.

Stereotactic electroencephalography was performed to diagnose the location of epileptogenicity. Five electrodes were implanted in the left and right temporal lobes. The first electrode was placed near the cavernous angioma. The second one was to the right anterior part of hippocampus. The third one was to the right posterior part of hippocampus. The fourth one was to the left anterior part of hippocampus. The fifth one was to the left posterior part of hippocampus. Ictal EEG showed that the patient’s seizures originated from the first electrode, spread to the right hippocampus, and later propagated to the left side. Consequently, the seizures did not originate from the hippocampus.

### 2:33 Surgical Strategy.

We chose to perform lesionectomy without hippocampectomy. Because the lesion was localized at the tip of the temporal lobe, it could be directly approached via the orbit to avoid any unnecessary brain damage with a small orbitotomy and craniotomy. The use of an endoscope provides a wide field of view in deeper part. On the other hand, conventional transcranial approach requires partial resection of the temporal lobe. Therefore, we selected the endoscopic transorbital approach.

### 3:08 Surgical Procedure.

The patient was placed in the supine position. An eyebrow skin incision was then made. The orbital rim was exposed and dissected under the periosteum. The periorbita was dissected from the orbital rim. The temporal muscle on the lateral side of the orbital rim was dissected and retracted. A partial orbitotomy was performed, and the orbital rim was removed. A 0° rigid endoscope was inserted. We preserved the orbital periosteum. The temporal muscle around the tip of the middle fossa was peeled off and retracted. The sphenoid bone was drilled to expose the dura mater. The dura mater around the sylvian fissure was exposed. A dural incision was then made. The arachnoid surface of the temporal lobe was identified. The surrounding hemosiderin deposition was confirmed, and a cavernous angioma was identified. Sufficient proximal bone drilling allowed sufficient space for endoscopic procedures. The cavernous angioma was removed and the surrounding hemosiderin deposits were aspirated. We identified the depth electrode at the deepest part, revealing that we had reached the posterior limit of the resection. The surrounding hemosiderin deposit was removed. The dural incision was approximately 1 cm. An artificial dural substitute was placed in an inlay and outlay manner for dural plasty. The bone defect was filled with abdominal fat tissue. The orbit was fixed using a titanium plate. The skin was sutured, and all procedures were completed.

### 5:22 Postoperative Course.

Postoperative MRI revealed complete removal of the lesion, including the surrounding hemosiderin deposit. The patient has been seizure free for 1 year after surgery without any complications.

### 5:36 Conclusions.

The endoscopic transorbital approach may be a less invasive surgical approach for temporal tip epileptogenic lesions.

## Disclosures

The authors report no conflict of interest concerning the materials or methods used in this study or the findings specified in this publication.

## Author Contributions

Primary surgeon: Uda, Tanoue. Assistant surgeon: Kawashima, Yindeedej. Editing and drafting the video and abstract: Tanoue. Critically revising the work: Uda. Reviewed submitted version of the work: Uda, Tanoue, Kawashima, Yindeedej. Approved the final version of the work on behalf of all authors: Uda. Supervision: Goto.

## Supplemental Information

### Patient Informed Consent

The necessary patient informed consent was obtained in this study.
